# Mashtree: a rapid comparison of whole genome sequence files

**DOI:** 10.21105/joss.01762

**Published:** 2019-12-10

**Authors:** Lee S. Katz, Taylor Griswold, Shatavia S. Morrison, Jason A. Caravas, Shaokang Zhang, Henk C. den Bakker, Xiangyu Deng, Heather A. Carleton

**Affiliations:** 1 Enteric Diseases Laboratory Branch, Centers for Disease Control and Prevention, Atlanta, GA, USA; 2 Center for Food Safety, University of Georgia, Griffin, GA, USA; 3 Respiratory Diseases Laboratory Branch, Centers for Disease Control and Prevention, Atlanta, GA, USA

## Summary

In the past decade, the number of publicly available bacterial genomes has increased dramatically. These genomes have been generated for impactful initiatives, especially in the field of genomic epidemiology (Brown, Dessai, McGarry, & Gerner-Smidt, 2019; Timme et al., 2017). Genomes are sequenced, shared publicly, and subsequently analyzed for phylogenetic relatedness. If two genomes of epidemiological interest are found to be related, further investigation might be prompted. However, comparing the multitudes of genomes for phylogenetic relatedness is computationally expensive and, with large numbers, laborious. Consequently, there are many strategies to reduce the complexity of the data for downstream analysis, especially using nucleotide stretches of length *k* (kmers).

One major kmer strategy is to reduce each genome to split kmers. With split kmer analysis, kmers on both sides of a variable site are recorded, and the variable nucleotide is identified. When comparing two or more genomes, the variable sites are compared. Split kmers have been implemented in software packages such as KSNP and SKA (Gardner, Slezak, & Hall, 2015; Harris, 2018).

Another major kmer strategy is to convert genomic data into manageable datasets, usually called sketches (Baker & Langmead, 2019; Ondov et al., 2016; Zhao, 2018). Most notably, an algorithm called min-hash was implemented in the Mash package (Ondov et al., 2016). In the min-hash algorithm, all kmers are recorded and transformed into integers using hashing and a Bloom filter (Bloom, 1970). These hashed kmers are sorted and only the first several kmers are retained. The kmers that appear at the top of the sorted list are collectively called the sketch. Any two sketches can be compared by counting how many hashed kmers they have in common.

Because min-hash creates distances between any two genomes, min-hash values can be used to rapidly cluster genomes into trees using the neighbor-joining algorithm (Saitou & Nei, 1987). We implemented this idea in software called Mashtree, which quickly and efficiently generates large trees that would be too computationally intensive using other methods.

## Implementation

### Workflow

**Figure 1:**
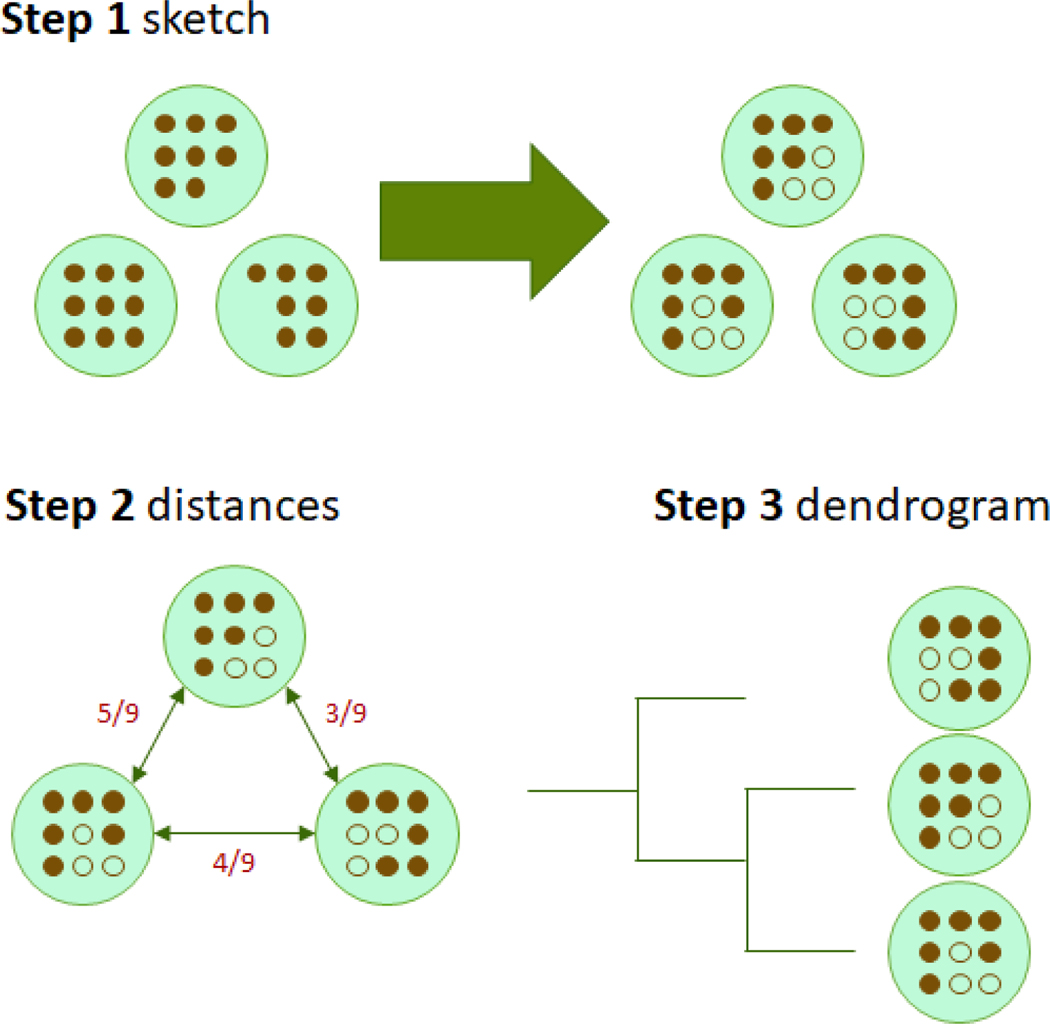
The Mashtree workflow. Step 1) Sketch genomes with Mash. In this schematic, there is a green circle representing each genome in the analysis. Filled-in brown circles indicate the presence of a kmer. Missing circles represent true absence. After hashing with a sketch size of six (after the arrow), some kmers are not represented in the Mash sketch either because they are not present in the original genome or because only a finite number of kmers are sketched (e.g., six in this example). Henceforth, truly missing hashes or hashes not included in the Mash sketch are represented by empty circles. Step 2) Calculate distances with Mash dist. Distances in the figure are represented by Jaccard distances, which are calculated as the intersection divided by the union. In this example, the genomes are separated by Jaccard distances of 5/9, 4/9, and 3/9. These Jaccard distances are internally transformed into Mash distances (Ondov et al., 2016). Step 3) Create dendrogram with Quicktree using the Mash distance matrix.

Mashtree builds on two major algorithms that are already implemented in other software packages. The first is the min-hash algorithm, which is implemented in the software Mash (Ondov et al., 2016). Mashtree uses Mash to create sketches of the genomes with the function mash sketch. We elected to keep most default Mash parameters but increased the sketch size (number of hashed kmers) from 1,000 to 10,000 to increase discriminatory power. Then, Mash is used to calculate the distances between genomes with mash dist. Mashtree records these distances into a pairwise distance matrix. Next, Mashtree calls the neighbor-joining (NJ) algorithm which is implemented in the software QuickTree (Howe, Bateman, & Durbin, 2002). The Mash distance matrix is used with QuickTree with default options to generate a dendrogram. The workflow is depicted in Figure 1.

### Confidence values

**Figure 2:**
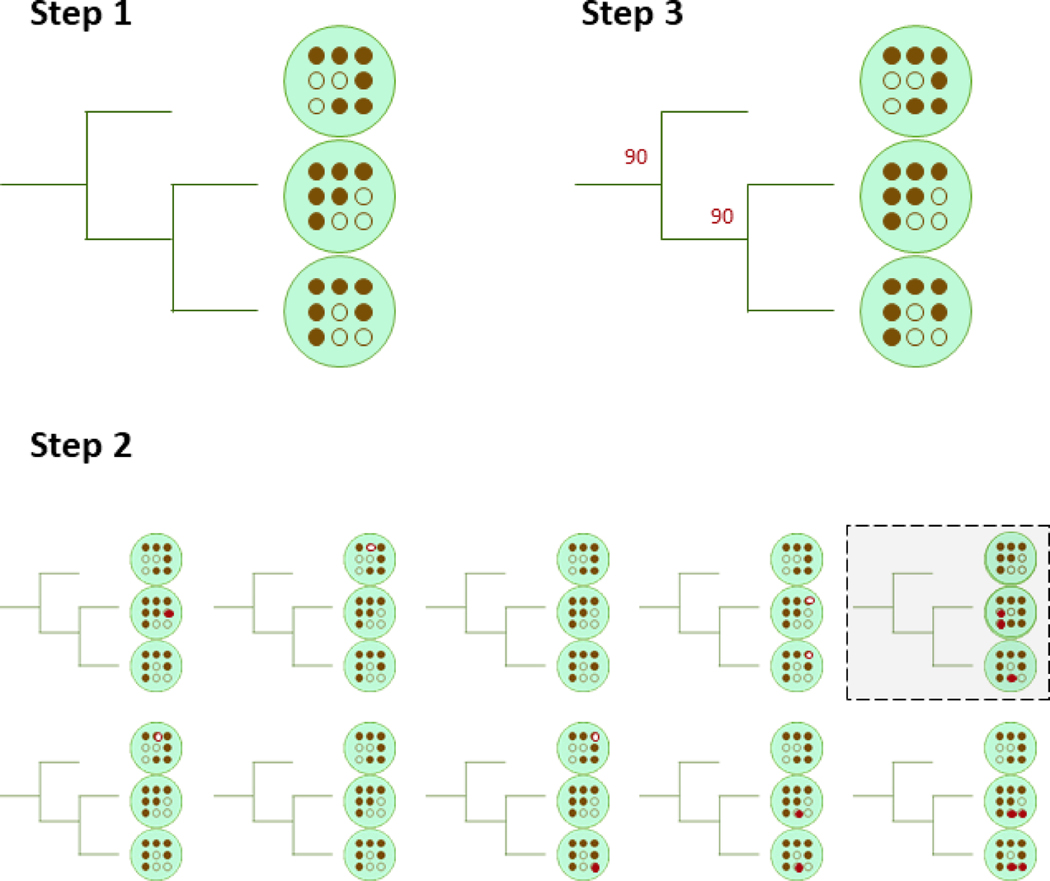
The Mashtree bootstrap workflow. Step 1) Generate a tree with the normal workflow as in Figure 1. This is the main tree. Step 2) Run the normal workflow once per replicate but with a different random seed. In this example, the top right replicate differs from the main tree. All ten of these trees are the bootstrap tree replicates. Step 3) For each parent node in the main tree, quantify how many bootstrap tree replicates have the same node with the same children. Record that percentage next to each parent node. This percentage quantifies how confident the Mashtree cluster is, controlling for the random seed in the Mash program.

**Figure 3:**
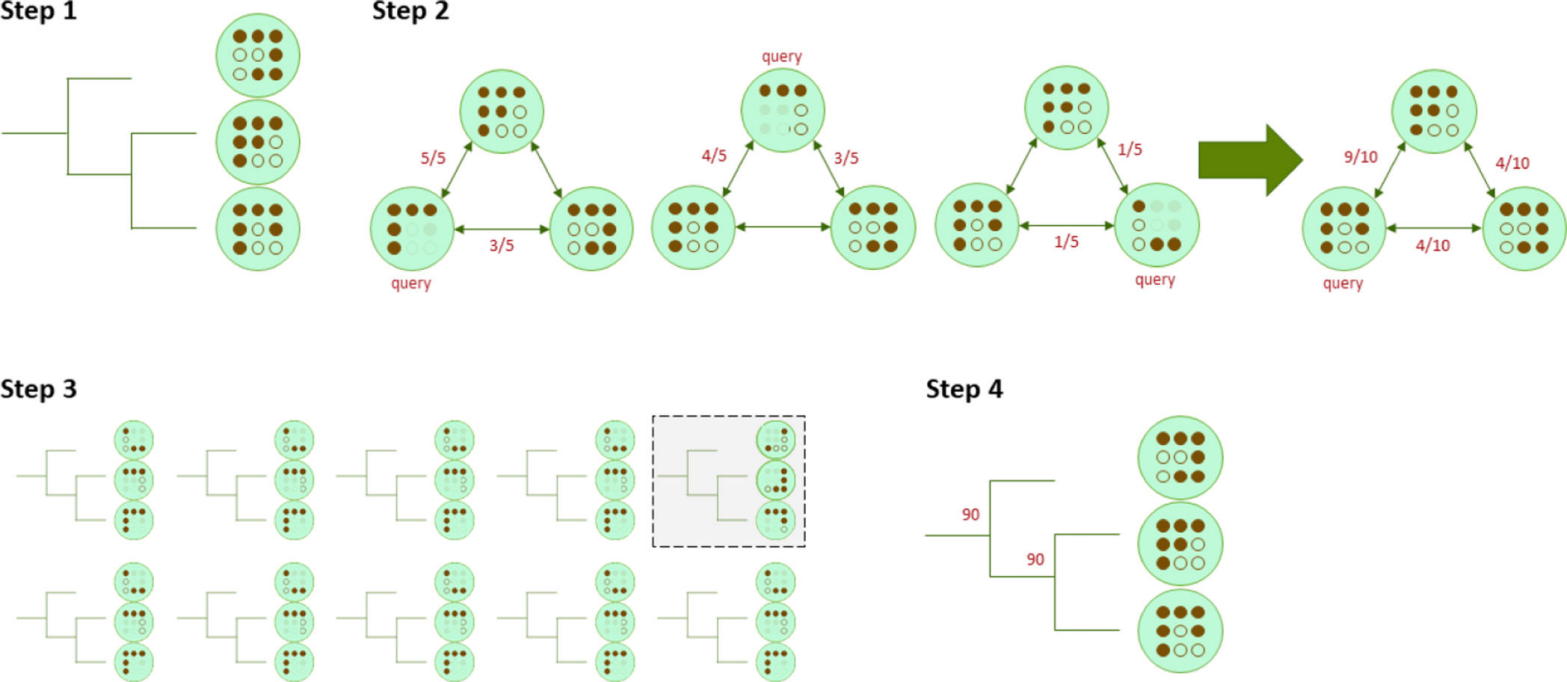
The Mashtree jackknife workflow. Step 1) Generate a tree with the normal workflow as in Figure 1. This is the main tree. Step 2) For each replicate, sample the half hashes without replacement for each query genome. Recalculate the Mash distance between the query genome and all other genomes, reducing the denominator to one half, rounding up, to reflect the smaller pool of hashes. After all genomes have been selected for query genomes, average the distances to create a new distance matrix. Create the dendrogram from the new distance matrix. For brevity, only one detailed replicate is shown. Step 3) For each replication, calculate the new tree from the new distance matrix. In this example, the top right replication differs from the main tree. All ten of these trees are the jackknife tree replicates. Step 4) For each parent node in the main tree, quantify how many jackknife tree replicates have the same node with the same children. Record that percentage next to each parent node. This percentage quantifies how confident Mashtree is at clustering, controlling for stochasticity in hashes.

Although Mashtree does not infer phylogeny, we have borrowed the ideas behind phylogenetic confidence values to yield confidence values for each parent node in the tree. There are two resampling methods implemented in Mashtree to assign support values to internal nodes: bootstrapping and jackknifing. Initially, both methods create a tree as depicted in Figure 1. Then, confidence values can be calculated for the tree using either the bootstrapping approach or the jackknifing approach (Figures 2 and 3).

### Other features

Mashtree has several other useful features. First, Mashtree can read any common sequence file type and can read gzip-compressed files (e.g., fastq, fastq.gz, fasta). This is a major advantage in being compatible with a wide variety of databases and with space-saving file compression. Second, Mashtree takes advantage of multithreading. The number of requested threads is used to determine how many genomes are sketched at the same time and how many sketches can be compared at the same time. When the number of threads requested outnumbers the number of operations that it can parallelize, Mashtree uses the multithreading already encoded in Mash sketches and distances. Third, Mashtree uses an SQLite database which can be used to cache results between runs.

## Installation

The Mashtree package is programmed in Perl, and is available in the CPAN repository. Documentation can be found at https://github.com/lskatz/mashtree.
